# Golden bifid might improve diarrhea-predominant irritable bowel syndrome via microbiota modulation

**DOI:** 10.1186/s41043-022-00302-0

**Published:** 2022-05-16

**Authors:** Mei Luo, Qin Liu, Lin Xiao, Li-Shou Xiong

**Affiliations:** Department of Gastroenterology and Hepatology, The First Affiliated Hospital of Sun Yat-Sen University, Guangzhou, 510080 China

**Keywords:** Fecal microbiota, Golden bifid, Self-Rating Anxiety Scale, Self-Rating Depression Scale, 16S rRNA gene-targeted pyrosequencing

## Abstract

**Objective:**

Gut microbiota might play a crucial role in the pathogenesis of irritable bowel syndrome (IBS), and probiotics supplement may be an effective treatment option. This study aims to explore the therapeutic effects of Golden bifid on the diarrhea-predominant IBS (IBS-D).

**Methods:**

Twenty-one consecutive IBS-D patients were recruited based on Rome IV criteria. All patients took 2000 mg Golden bifid triple daily for 4 weeks. Gastrointestinal (GI) symptoms, psychological symptoms, small intestine bacterial overgrowth (SIBO) and fecal microbiota characteristics were evaluated in IBS-D patients before and after treatment.

**Results:**

After 4-week treatment of Golden bifid, the GI symptoms such as abdominal pain (2.90 ± 1.04 vs. 1.90 ± 1.26, *P* = 0.002), abdominal distension (2.00 ± 1.34 vs. 1.29 ± 1.31, *P* = 0.007), diarrhea (3.24 ± 1.37 vs. 1.81 ± 1.21, *P* = 0.001), defecatory urgency (3.48 ± 1.03 vs. 2.33 ± 1.35, *P* = 0.000) and incomplete evacuation (2.71 ± 1.15 vs. 1.76 ± 1.26, *P* = 0.003) were significantly alleviated in IBS-D patients. The Self-Rating Depression Scale (SDS) decreased significantly (46.19 ± 11.36 vs. 43.33 ± 9.65, *P* = 0.041), and SIBO could be eradicated in 25% (4/16) of IBS-D patients with SIBO. Meanwhile, the abundance of *Unclassified Lachnospiraceae* and *Dorea* in genus level and *Unclassified Lachnospiraceae*, *Bacterium Dorea*, *Bacterium Butyricicoccus* and *Dorea formicigenerans ATCC 27755* in species level were increased in fecal microbiota (*P* < 0.05).

**Conclusions:**

Golden bifid could improve most of GI symptoms and depressive symptoms in IBS-D patients and eradicate a small proportion of SIBO in those IBS-D patients with SIBO. What's more, Golden bifid could also modulate the fecal microbiota in IBS-D patients, which implied that the Golden bifid might improve IBS-D via microbiota modulation.

**Supplementary Information:**

The online version contains supplementary material available at 10.1186/s41043-022-00302-0.

## Introduction

Irritable bowel syndrome (IBS) is one of the most common globally functional bowel disorders (FBDs). The characteristics of IBS include abdominal pain, abdominal distension, changes in defecation habits, insignificant morphology and biochemics, and its pathogenesis is unclear now [1]. The global prevalence of IBS is as high as 3.3–31.6% with an average prevalence of 8.8% [2], and about 6.5% in China [3]. Although the pathogenesis of IBS is unclear, gut microbiota dysbiosis was found to be associated with the pathogenesis of IBS [4].

The alterations of gut microbiota could damage the intestinal mucosal barrier function, intestinal permeability, immunity, releasing histamine, 5-hydroxytryptamine, prostaglandins and tryptase and other active substances. The microbiota-derived metabolites eventually cause abdominal pain, diarrhea and other intestinal symptoms [5–8]. Therefore, probiotics intervention has become a significant therapeutic method of IBS for gut microbiota disorder in recent years. Probiotics treatment might be a novel approach to improve the intestinal epithelial barrier function, inhibit the adhesion of pathogenic bacteria and mediate the regulation of immune system. Moreover, research have shown that probiotics therapy could modulate the gut microbiota of IBS patients [9]. *Bifidobacterium bifidum* could effectively alleviate IBS and improve IBS symptoms, including abdominal pain, discomfort, bloating, urgency and digestive disorder simultaneously with an improvement of quality of life [10]. After probiotics treatment, the number of *Lactobacillus* and *Bifidobacterium* in the feces of IBS patients increased significantly [9]. Meanwhile, Ma et al*.* [11] put forwarded that probiotics supplement might improve the psychological symptoms of IBS patients by regulating the balance of gut microbiota and gut–brain axis. Small intestinal bacterial overgrowth (SIBO) is a clinical syndrome that changes the characteristic or quantity of bacteria in the small intestine. Lactulose hydrogen breath test (LHBT) is a safe, convenient, efficient and noninvasive diagnostic method for SIBO. Study has found that SIBO was one of the pathogenesises of IBS, as eradicating SIBO could alleviate the symptoms of IBS patients [12].

Although some studies have shown that probiotic treatment could improve the IBS and regulate the gut microbiota disorders, there is no consensus on its therapeutic effect and mechanisms. The purpose of this study was to evaluate whether Golden bifid treatment could modulate the fecal microbiota of IBS-D patients by 16S rRNA gene-targeted pyrosequencing technology and explore its therapeutic effect on GI symptoms, SIBO eradication rate and psychological problems of IBS-D patients.

## Methods

### Study subjects

IBS-D patients were recruited from the Department of Gastroenterology and Hepatology, The First Affiliated Hospital of Sun Yat-sen University, from June 2019 to December 2020. All patients met Rome IV criteria and aged between 18 and 65. They were required to perform a colonoscopy to exclude organic diseases. The protocol was approved by the Medical Ethics Committee of the First Affiliated Hospital of Sun Yat-sen University, and all patients had signed informed consent.

### Study design and procedure

All patients took 2000 mg Golden bifid (INNER MONGOLIA Shuangqi Pharmaceutical Co., Ltd., Inner Mongolia, China. 1 × 10^7^ CFU/g *Bifidobacterium Longum,* 1 × 10^6^ CFU/g *Lactobacillus bulgaricus* and 1 × 10^6^ CFU/g *Streptococcus thermophilus*) triple daily for 4 weeks. One fasting fecal sample was collected from all patients for 16S rRNA gene-targeted pyrosequencing at baseline (T0) and the end of treatment (T28) to evaluate the characteristics of gut microbiota. Meantime, all patients completed GI symptom severity scale, Self-Rating Anxiety Scale (SAS), Self-Rating Depression Scale (SDS) questionnaires and lactulose hydrogen breath test (LHBT).

### Small intestinal bacterial overgrowth

The LHBT was completed for all IBS-D patients according to a standard procedure. And according to our previous research [13], when the baseline value of H2 ≥ 20 p.p.m and/or a rise of > 20 p.p.m. from baseline in H2 within 90 min of lactulose administration, the LHBT should be considered a presence of SIBO.

### GI symptoms and psychological symptoms

Three questionnaires including GI symptom severity scale [13], SAS [14] and SDS [14] were performed in all IBS-D patients at T0 and T28. The questionnaire of GI symptom severity scale includes abdominal discomfort, abdominal distension, abdominal pain, diarrhea, defecatory urgency and incomplete evacuation, six GI symptoms with seven-point Likert responses, and the final score is negatively correlated with GI symptoms. The SAS and SDS contain 20 items with four-point Likert responses about anxiety and depression, respectively, and the final score is negatively correlated with anxiety or depression symptoms too.

### 16S rRNA gene-targeted pyrosequencing

#### Fecal sample collection and DNA extraction

Fecal samples were collected by germ-free disposable sampling spoon and frozen immediately at − 80 °C. Fecal microbial DNA was extracted from 180–220 mg feces using E.Z.N.A.^®^ soil Kit (Omega Bio-tek, Norcross, GA, USA) according to manufacturer’s protocols. The DNA concentration and purification were determined by NanoDrop 2000 UV–Vis spectrophotometer (Thermo Scientific, Wilmington, USA), and DNA quality was checked by 1% agarose gel electrophoresis. The qualified fecal bacterial genomic DNA samples were stored in Tris–HCl buffer, pH 8.0, at − 20 °C.

### PCR amplification

The V3-V4 hypervariable regions of the bacteria 16S rRNA gene were amplified with primers 338F (5′-ACTCCTACGGGAGGCAGCAG-3′) and 806R (5′-GGACTACHVGGGTWTCTAAT-3′) by thermocycler PCR system (GeneAmp 9700, ABI, USA). The PCR reactions were conducted using the following program: 3 min of denaturation at 95 °C, 27 cycles of 30 s at 95 °C, 30 s for annealing at 55 °C, and 45 s for elongation at 72 °C, and a final extension at 72 °C for 10 min. PCR reactions were performed in triplicate 20 μL mixture containing 4 μL of 5 × FastPfu Buffer, 2 μL of 2.5 mM dNTPs, 0.8 μL of each primer (5 μM), 0.4 μL of FastPfu Polymerase and 10 ng of template DNA. The resulted PCR products were extracted from a 2% agarose gel and further purified using the AxyPrep DNA Gel Extraction Kit (Axygen Biosciences, Union City, CA, USA) and quantified using QuantiFluor^™^-ST (Promega, USA) according to the manufacturer’s protocol.

### Illumina MiSeq sequencing

Purified amplicons were pooled in equimolar and paired-end sequenced (2 × 300) on an Illumina MiSeq platform (Major Bio-Pharm Technology, Shanghai, China) according to the standard protocols by Majorbio Bio-Pharm Technology Co. , Ltd. (Shanghai, China).

### Processing of sequencing data

Raw fastq files were demultiplexed, quality-filtered by Trimmomatic and merged by FLASH with the following criteria: (i) The reads were truncated at any site receiving an average quality score < 20 over a 50 bp sliding window. (ii) Primers were exactly matched allowing 2 nucleotide mismatching, and reads containing ambiguous bases were removed. (iii) Sequences whose overlap is longer than 10 bp were merged according to their overlap sequence. Operational taxonomic units (OTUs) were clustered with 97% similarity cutoff using UPARSE, and chimeric sequences were identified and removed using UCHIME. The taxonomy of each 16S rRNA gene sequence was analyzed by RDP Classifier algorithm against the Silva (SSU123) 16S rRNA database using confidence threshold of 70%.

### Microbiological analysis

Operational taxonomic units (OTUs) were clustered with 97% similarity cutoff using UPARSE version 7.1, and chimeric sequences were identified and removed using UCHIME. The taxonomy of each 16S rRNA gene sequence was analyzed by RDP Classifier algorithm against the Silva (138/16s_bacteria) 16S rRNA database using confidence threshold of 70%. Then, the rank abundance curves, rarefaction curves, Alpha-diversity analysis, Beta-diversity analysis and community bar chart were used to analyze and compare the characteristics of fecal microbiota in patients with IBS-D before and after Golden bifid treatment. The rank abundance curves were plotted to determine the species abundance and community evenness of each sample, and the range of the curve on the horizontal axis and the gentle degree of the curve on the vertical axis reflect the species abundance and community evenness of the sample, respectively. Rarefaction curves were drawn to determine whether the sample size is sufficient and sequencing data of each sample. Alpha-diversity analyses including community richness parameters (Sobs, Ace, Chao1), community diversity parameters (Shannon, Simpson) and community coverage parameters (Good’s coverage) were calculated using the Mothur software. Principal coordinates analysis (PCoA), which is a Beta-diversity analyses, was used to compare the community composition of different samples. Community composition analysis (community bar chart) was used to analyze the species composition drawn with R package software (version 3.3.1). In addition, bacterial taxonomic distributions of sample communities were visualized using the R package software. Microbiome features differences between two groups (before and after Golden bifid treatment) were analyzed with Wilcoxon signed-rank test. PICRUSt2 analysis (KEGG level) was used to predict the functional profiling of microbial communities [12, 15].

### Statistical analysis

SPSS 23.0 (SPSS, Inc., Chicago, IL, USA) and Graph Prism version 7.0 (GraphPad Software, Inc., La Jolla, CA, USA) were used for statistical analysis and chart making. The measurement data were expressed in mean differences ± standard deviation ($\overline{x} \pm s$). Paired *T* test or Wilcoxon test was used for comparison. For all results of statistical analysis, *P* < 0.05 was considered statistically significant and if *P* < 0.01, the difference was statistically significant.

## Results

### Study subjects

Twenty-one consecutive IBS-D patients (5F, 16 M; age 31.38 ± 8.31 year, BMI 21.06 ± 2.11 kg/m^2^) who met Rome IV diagnostic criteria, excluded organic diseases, fulfilled inclusion criteria and agreed to participate in this study were recruited in our study. Table 1 summarizes the demographic and clinical characteristics of all IBS-D patients at baseline.

**Table 1:** Demographic and clinical characteristics of IBS-D patients at baseline

	IBS-D (*n* = 21)
Variable	
Gender (*n*, %)	
Female	5 (23.81)
Male	16 (76.19)
Age, year (mean ± SD)	31.38 ± 8.31
BMI, kg/m^2^ (mean ± SD)	21.06 ± 2.11
Duration, year (*n*, %)	
< 1	0 (0)
1–3	7 (33.33)
4–10	9 (42.86)
11–20	3 (14.29)
> 20	2 (9.52)
LHBT^†^(*n*, %)	
LHBT (+)	16 (76.19)
LHBT (−)	5 (23.81)
Symptoms score (mean ± SD)	
Abdominal pain	2.90 ± 1.04
Abdominal distension	2.00 ± 1.34
Abdominal discomfort	2.29 ± 1.19
Diarrhea	3.24 ± 1.37
Defecatory urgency	3.48 ± 1.03
Incomplete evacuation	2.71 ± 1.15
SAS^‡^	44.43 ± 6.38
SDS^§^	46.19 ± 11.36

^†^*LHBT* Lactulose hydrogen breath test

^‡^*SAS* Self-Rating Anxiety Scale

^§^*SDS* Self-Rating Depression Scale

### Lactulose hydrogen breath test

After a LHBT at T0, sixteen IBS-D patients had a positive LHBT (six patients have a baseline value of H2 ≥ 20 p.p.m, while the rest have a rise of > 20 p.p.m from baseline in H2 within 90 min of lactulose administration) and were considered a presence of SIBO. After 4-week treatment with Golden bifid, 4 of the 16 (25%) patients with SIBO had a negative LHBT, which implied that the SIBO could be partially eradicated by Golden bifid.

### GI symptoms and psychological symptoms

After a 4-week treatment of Golden bifid, the GI symptoms in IBS-D patients including abdominal pain (2.90 ± 1.04 vs. 1.90 ± 1.26), abdominal distension (2.00 ± 1.34 vs. 1.29 ± 1.31), diarrhea (3.24 ± 1.37 vs. 1.81 ± 1.21), defecatory urgency (3.48 ± 1.03 vs. 2.33 ± 1.35) and incomplete evacuation (2.71 ± 1.15 vs. 1.76 ± 1.26) were improved significantly (*P* < 0.05) (Table 2). In psychological symptoms, the SDS was decreased significantly (46.19 ± 11.36 vs. 43.33 ± 9.65, *P* < 0.05), while there was no significant difference in SAS (Table 2).

**Table 2:** GI symptoms scores and psychological symptoms scores of IBS-D patients: pre-treatment and post-treatment

Symptoms	Pre-treatment	Post-treatment	*P* value
Abdominal pain	2.90 ± 1.04	1.90 ± 1.26	0.002*
Abdominal distension	2.00 ± 1.34	1.29 ± 1.31	0.007*
Abdominal discomfort	2.29 ± 1.19	1.86 ± 1.06	0.140
Diarrhea	3.24 ± 1.37	1.81 ± 1.21	0.001*
Defecatory urgency	3.48 ± 1.03	2.33 ± 1.35	0.000*
Incomplete evacuation	2.71 ± 1.15	1.76 ± 1.26	0.003*
SAS^†^	44.43 ± 6.38	43.71 ± 6.54	0.485
SDS^‡^	46.19 ± 11.36	43.33 ± 9.65	0.041*

^†^*SAS* Self-Rating Anxiety Scale

^‡^*SDS* Self-Rating Depression Scale

*P < 0.05 was considered statistically significant

### Characterization of gut microbiota in IBS-D

A total of 42 fecal samples of 21 IBS-D patients before and after Golden bifid treatment were analyzed by 16S rRNA gene-targeted pyrosequencing to observe and compare the characterization of fecal microbiota in IBS-D patients at T0 and T28. After demultiplexing and quality-filtering the original sequencing data, 2,046,464 high-quality sequences were obtained with an average of 48,725 sequences per sample. The sequences with 97% similarity cutoff were clustered, and 715 OTUs were obtained.

**Fig. 1:**
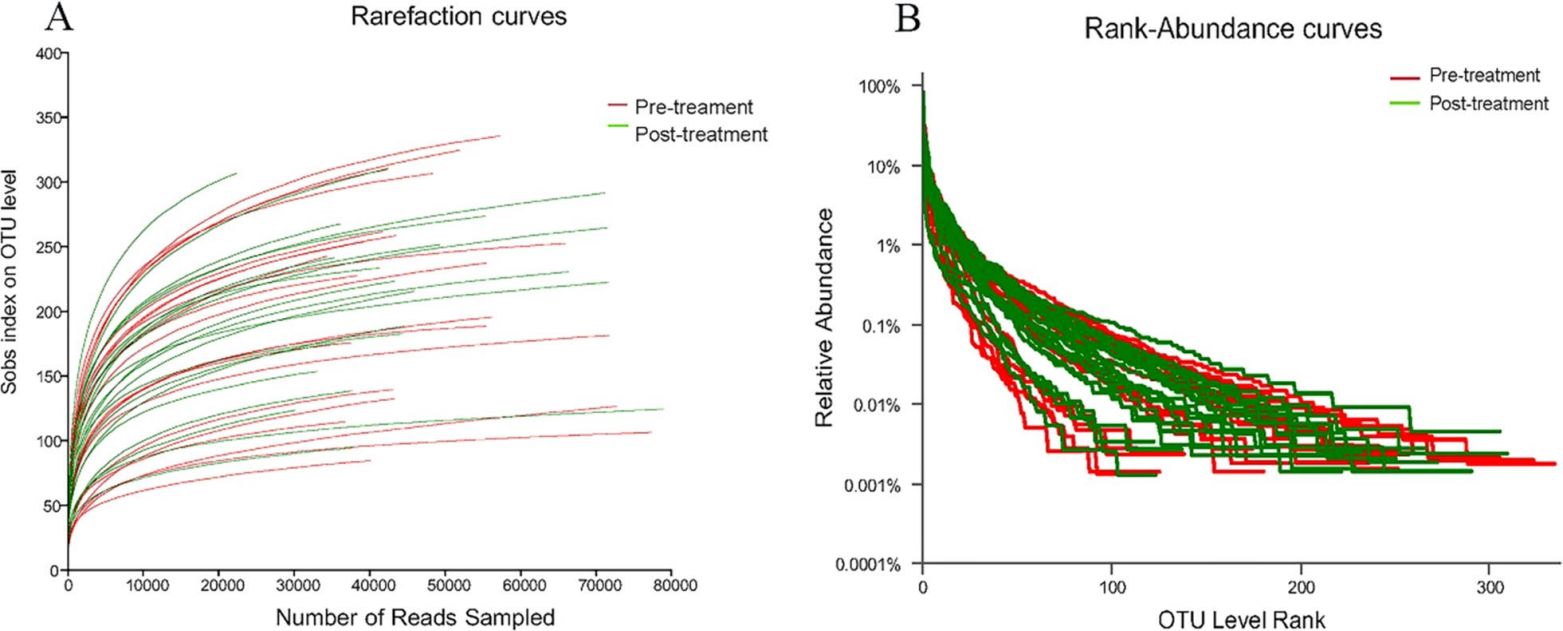
Sequencing depth of the 42 fecal samples in IBS-D patients before and after Golden bifid treatment. **A** Rarefaction curves. **B** Rank abundance curves

The rarefaction curve (Fig. 1A) and rank abundance curves (Fig. 1B) for the 42 samples generated using R software were clustered at the 97% phylotype similarity level. Thus, the sequencing depth was sufficient for exploring the microbial community and the species abundance, and community evenness was suitable for further analysis.

The fecal microbiota richness, evenness and community coverage before and after Golden bifid treatment of IBS-D patients are shown in Table 3. No significant differences in the richness parameters (Sobs, Ace, Chao1) and community diversity parameters (Shannon, Simpson) (*P* > 0.05) were found in IBS-D patients before and after treatment. Meanwhile, the sequencing depth was sufficient for analyzing fecal microbiota according to the coverage parameters values (> 99.9%).

**Table 3:** Fecal microbiota community indices before and after treatment

Community indices	Pre-treatment (mean)	Pre-treatment (SD^†^)	Post-treatment (mean)	Post-treatment (SD)	*P* value
Sobs	214.43	62.752	211.62	75.831	0.8966
Ace	246.15	67.273	245.68	82.401	0.9841
Chao 1	249.03	69.702	240.49	82.558	0.7192
Shannon	3.1523	0.71986	3.0933	0.74728	0.7959
Simpson	0.12578	0.16176	0.12593	0.13854	0.9975
Coverage	0.99913	0.000425	0.99926	0.000341	0.2688

^†^*SD* standard deviation

**Fig. 2:**
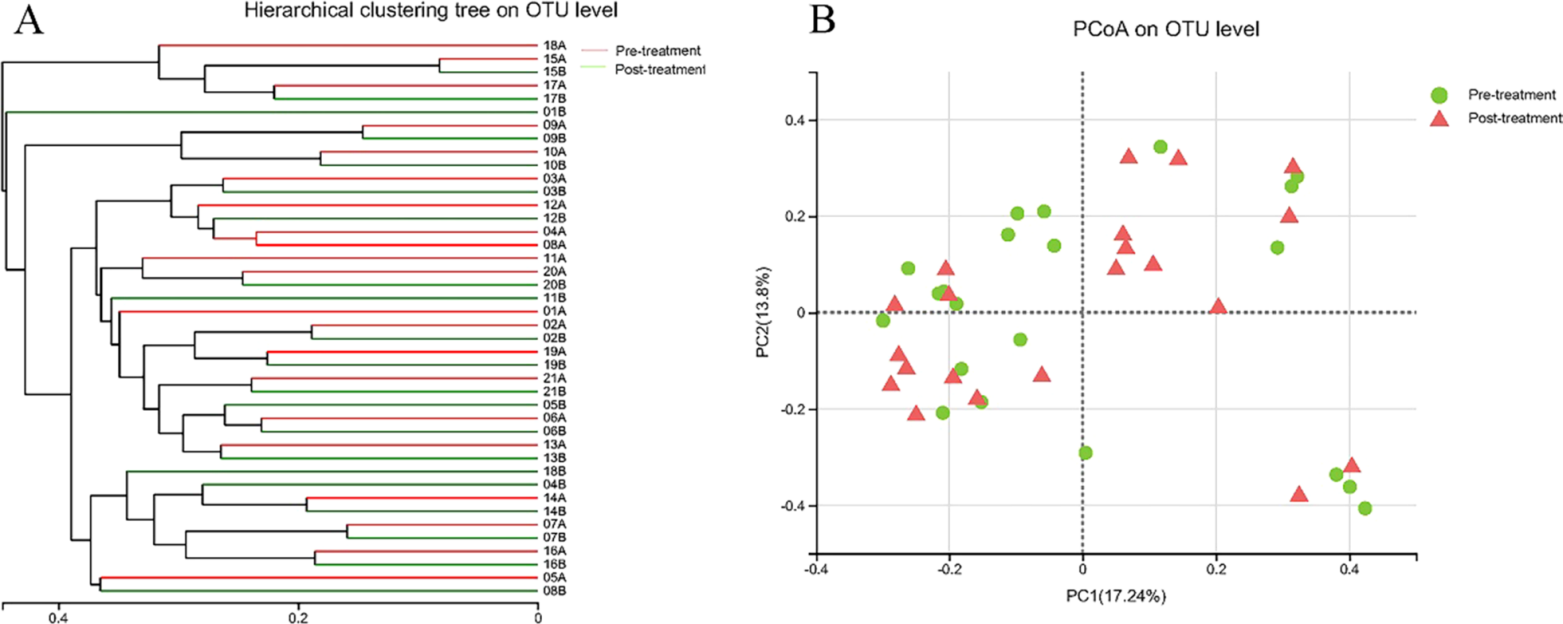
Structural comparison of fecal microbiota from IBS-D patients before and after Golden bifid treatment. **A** Hierarchical clustering analysis of fecal microbiota in IBS-D patients before and after Golden bifid treatment. Each branch and color on the tree represented one sample and one group (the IBS-D patients before and after Golden bifid treatment), respectively. **B** Principal coordinates analysis (PCoA) of fecal microbiota in IBS-D patients before and after Golden bifid treatment

Hierarchical clustering dendrogram and PCoA were used to analyze the similarity between microbial communities in the 42 fecal samples (Fig. 2). The overall trend of the hierarchical clustering dendrogram was that gut microbiota of one IBS-D patients before and after Golden bifid treatment clustered together firstly and then clustered with other patients, which indicated that the fecal microbiota from IBS-D patients before and after Golden bifid treatment could not be divided into two different clusters completely (Fig. 2A). Similarly, on the PCoA graph, the fecal microbiota in IBS-D patients before and after treatment could not be separated clearly into two different clusters (Fig. 2B**)**.

**Fig. 3:**
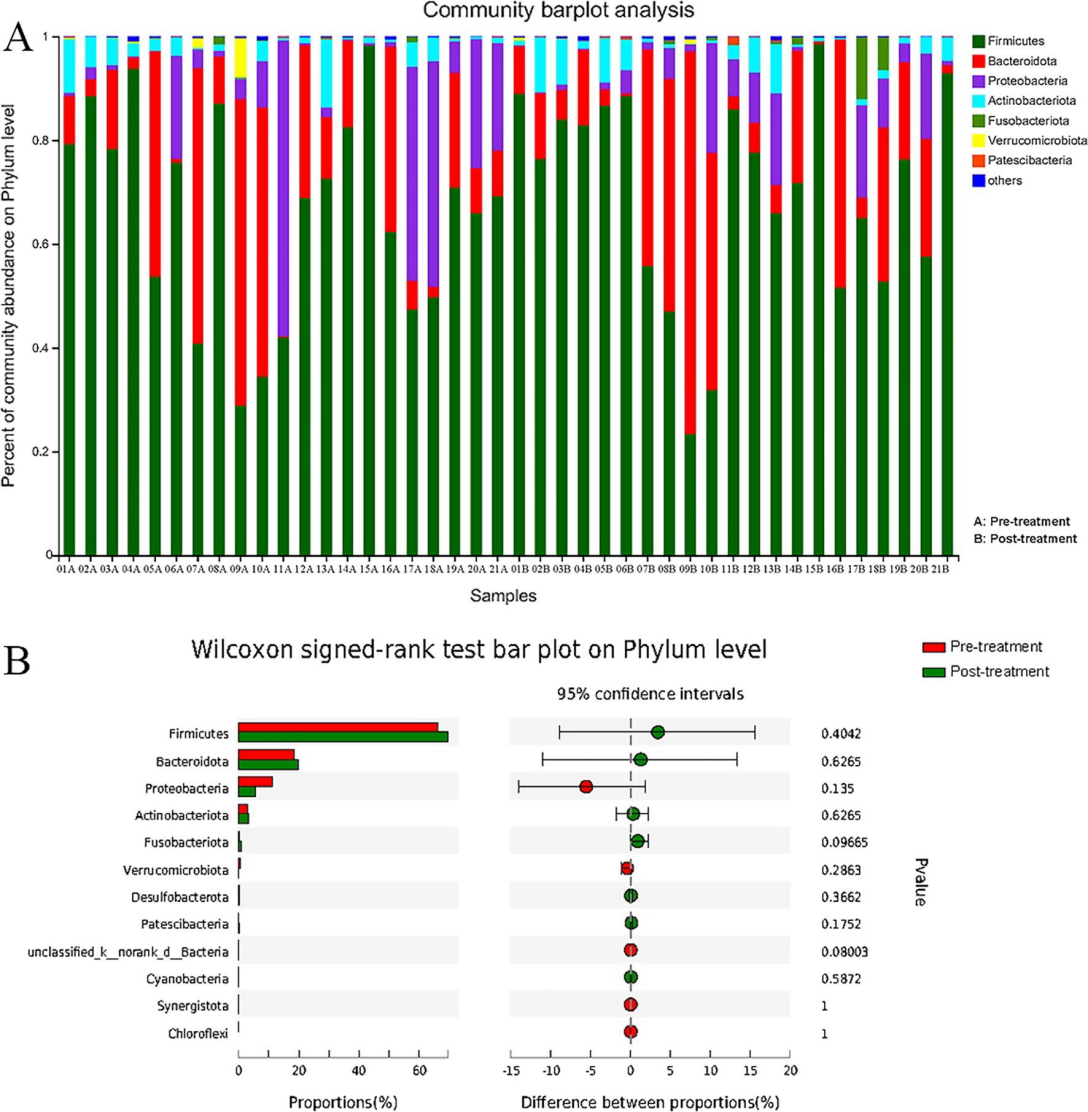
Characteristics of fecal microbiota at the phylum level. **A** Relative abundance of fecal microbiota at the phylum levels in IBS-D patients before and after Golden bifid treatment. **B** Comparison of relative abundance of fecal microbiota at phylum levels in IBS-D patients before and after Golden bifid treatment

RDP classifier was used to analyze the taxonomy of microbiota in the 42 fecal samples. At the phylum level, twelve phyla were found in all fecal samples. As shown in Fig. 3, the composition of fecal microbiota in each sample was different in phylum level (Fig. 3A). There was no significant difference of fecal microbiota at phylum level in IBS-D patients before and after Golden bifid treatment (Fig. 3B).

**Fig. 4:**
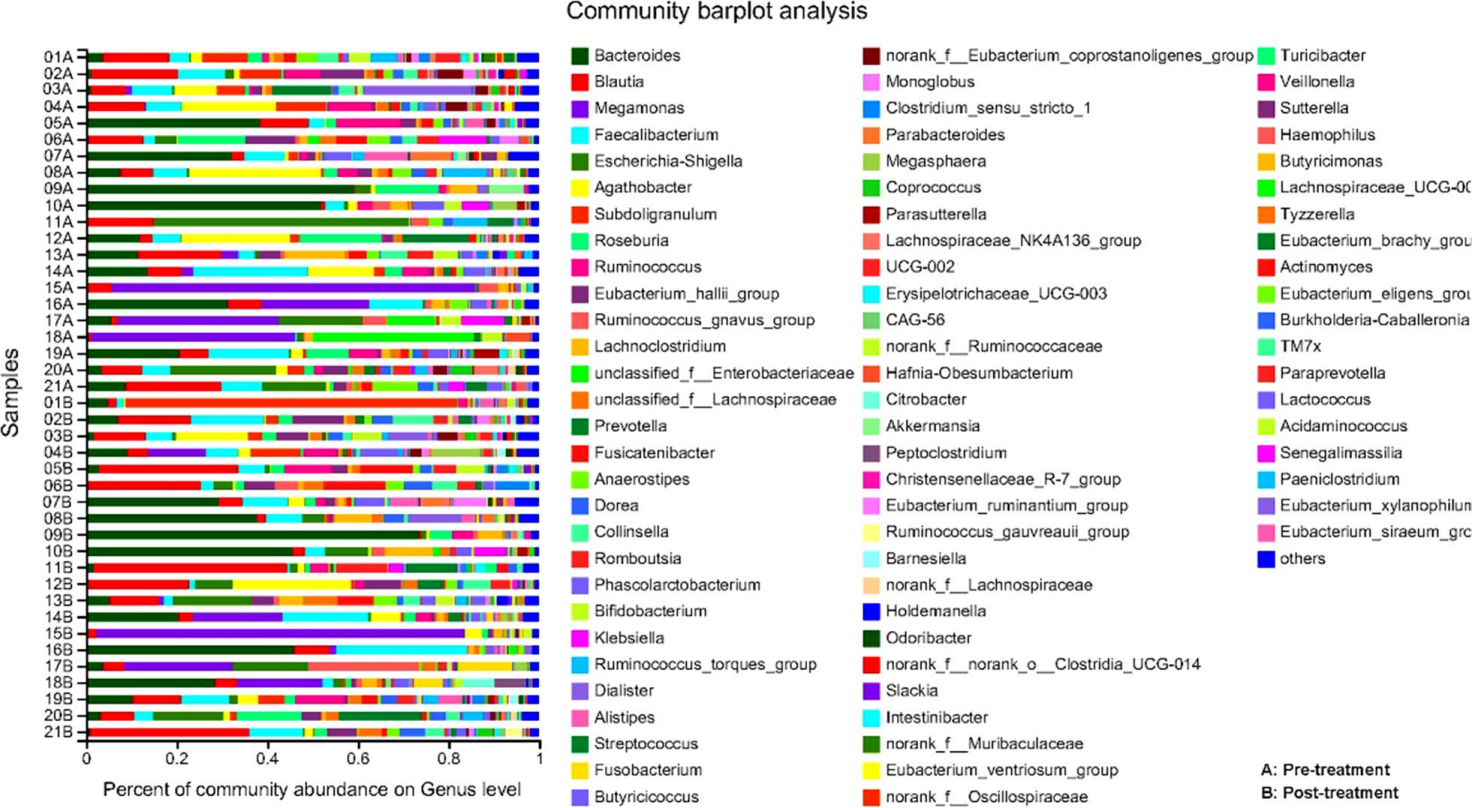
Characteristics of fecal microbiota at the genus level. Relative abundance of fecal microbiota at the genus levels in IBS-D patients before and after Golden bifid treatment

At the genus levels, 257 genera were found in the 42 fecal samples. *Bacteroides*, *Blautia*, *Megamonas*, *Faecalibacterium* and *Escherichia-shigella* were the most predominant genera in fecal samples from IBS-D patients before and after Golden bifid treatment (Fig. 4). After treatment, the abundance of *Unclassified Lachnospiraceae* and *Dorea* were increased in fecal microbiota of IBS-D patients, while the *Ruminococcaceae NK4A214 group*, *unclassified Ruminococcaceae*, Coriobacteriales Incertae Sedis and *Peptoniphilus* were decreased (*P* < 0.05) (Table 4, Additional file 1: Fig. S1A). And at the species levels, we found that the abundance of *Bacteroides xylanisolvens Bacteroides*, *Unclassified Lachnospiraceae*, *Bacterium Dorea*, *Bacterium Butyricicoccus*, *Dorea formicigenerans ATCC 27755*, *Unclassified Fusobacterium* and *Unclassified Bifidobacterium* were increased in feces, while the *Unclassified NK4A214 group*, *Unclassified Ruminococcaceae*, *Gut metagenome Roseburia*, *Unclassified Oscillibacter* and *Uncultured organism Oribacterium* were decreased (*P* < 0.05) (Table 4, Additional file 1: Fig. S1B).

**Table 4:** Fecal microbiota in IBS-D patients before and after treatment

Taxon	Pre-treatment (mean)	Pre-treatment (SD^†^)	Post-treatment (mean)	Post-treatment (SD)	*P* value
Genus					
*Unclassified Lachnospiraceae*	1.298	1.024	2.124	1.827	0.02854
*Dorea*	1.199	1.196	1.88	2.095	0.01987
* Ruminococcaceae NK4A214 group*	0.146	0.2565	0.09111	0.1912	0.01673
*Unclassified Ruminococcaceae*	0.1359	0.232	0.05778	0.09483	0.01661
Coriobacteriales Incertae Sedis	0.02911	0.05526	0.006373	0.009627	0.04401
*Peptoniphilus*	0.002012	0.006217	6.69E-05	0.000307	0.01427
Species					
*Bacteroides xylanisolvens Bacteroides*	1.588	2.525	2.697	5.52	0.04381
*Unclassified Lachnospiraceae*	1.298	1.024	2.124	1.827	0.02854
*Bacterium Dorea*	0.9228	1.063	1.388	1.973	0.0403
*Bacterium Butyricicoccus*	0.6035	0.9297	0.7092	0.7979	0.04189
*Dorea formicigenerans ATCC 27755*	0.2761	0.3662	0.4921	0.7504	0.04067
*Unclassified Fusobacterium*	0.0131	0.02308	0.356	1.197	0.04149
*Unclassified NK4A214 group*	0.1452	0.2544	0.08982	0.1887	0.01847
*Unclassified Ruminococcaceae*	0.1359	0.232	0.05778	0.09483	0.01661
*Gut metagenome Roseburia*	0.03581	0.145	0.01651	0.07418	0.03603
*Unclassified Oscillibacter*	0.01026	0.01981	0.003806	0.009182	0.01906
*Uncultured organism Oribacterium*	0.003345	0.003436	0.003265	0.009434	0.03281
*Unclassified Bifidobacterium*	0	0	0.005316	0.01523	0.03603

^†^*SD* standard deviation

**Fig. 5:**
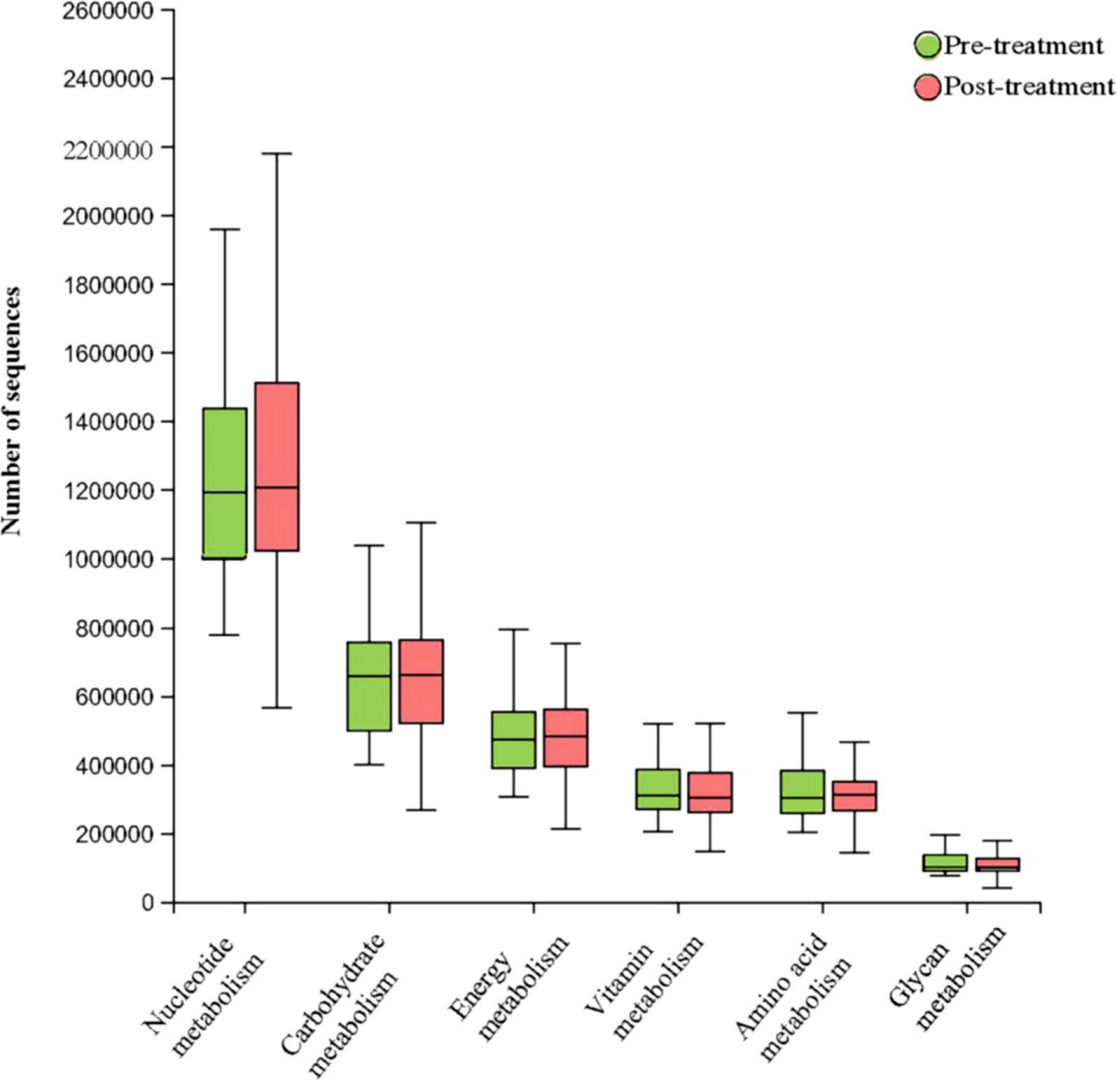
Basic metabolism pathways of gut microbiome in IBS-D patients before and after Golden bifid treatment

We found that there were some alterations of microbial community structure in IBS-D patients after Golden bifid treatment at the genus and species level. To explore the potential role of these changes in fecal microbial compositions, we used PICRUSt2 to infer microbial gene content from 16S rRNA gene data and aggregated relative abundance of functional genes into metabolic pathways. We have analyzed the basic metabolism pathways of gut microbiome including carbohydrate metabolism, amino acid metabolism, nucleotide metabolism, energy metabolism, vitamins metabolism and glycan metabolism **(**Fig. 5**)**. However, no significant differences of these metabolites were found in IBS-D patients before and after treatment.

### Adverse events

No deaths and other adverse events occurred during Golden Bifid treatment. Therefore, the treatment of Golden Bifid was well tolerated.

## Discussion

In this study, we found that 4-week treatment of Golden bifid could not only alleviate the symptoms of abdominal pain, abdominal distension, diarrhea, defecatory urgency and incomplete evacuation, but also improve the depressive symptoms and modulate the fecal microbiota of IBS-D patients. Thus, we inferred that the Golden bifid might improve the symptoms of IBS-D by intervening gut microbiota.

Many studies have shown that the alterations of gut microbiota might be highly associated with IBS [12, 16]. A systematic review showed that the *Enterobacteriaceae*, *Lactobacillaceae* and *Bacteroides* were increased, while *uncultured Clostridiales*, *Faecalibacterium* and *Bifidobacterium* were decreased in IBS patients compared with healthy controls [17]. Therefore, the manipulation of the microbiota and probiotics treatments is becoming therapeutic option for IBS [18] and the effectiveness of probiotics might be different due to the strain and dose-specific [19]. In our study, we found that the GI symptoms including abdominal pain, abdominal distension, diarrhea, defecatory urgency and incomplete evacuation of IBS-D patients were improved significantly after treatment. Hoveyda et al. [20] also showed that probiotics could effectively alleviate the overall symptom score of IBS patients. Although the therapeutic mechanism of Golden bifid is unclear, it might be related to the improvement of intestinal epithelial barrier function, relief of visceral hypersensitivity, inhibition of adhesion pathogenic bacteria and regulation of the immune system. Probiotics intervention could increase the expression of tight junction protein, reduce the levels of interferon and interleukin-4 (IL-4) in colonic mucosa and improve the intestinal barrier function [21], which might relieve the symptom of diarrhea. In addition, *Bifidobacterium longum* alleviated visceral hypersensitivity in rats by enhancing lysozyme production and promoting mucosal repair and result in the relief of abdominal pain [22].

With the development of the viewpoint of brain–gut–microbiota axis, psychological factors are also considered to be a pathogenesis of IBS and abnormalities in gut microbiota can influence changes in psychological health [23]. In this study, we found that the depression scores in IBS-D patients were reduced by probiotic Golden bifid. Similarly, Ma et al. [11] supported that probiotic could improve the mental well-being of IBS and this improvement was possibly mediated by restoration of microbial balance and the brain–gut axis. Probiotics could not only normalize noradrenaline levels in the brainstem region that regulates emotion, but also alter other brain neurotransmitters, such as gamma-aminobutyric acid and serotonin [24, 25], which might be the influencing factors to mental health.

In addition, we observed that a small proportion SIBO could be eradicated in those IBS-D patients with SIBO after Golden bifid treatment, which might imply that Golden bifid could eradicate SIBO by modulating the gut microbiota. Research showed that the abundances of *Lactobacillus* and *Bifidobacterium* in the feces of IBS patients were increased significantly after probiotics intervention [9]. *Bifidobacterium* could increase IgA antibody, provide an immune barrier to mucosal surface, enhance the function of the immune system and ultimately improve the disease resistance of human body [26]. Similarly, the results of 16S rRNA gene-targeted pyrosequencing in our study showed that there were some alterations of fecal microbiota after Golden bifid treatment. At the genus level, the abundance of *Unclassified Lachnospiraceae* and *Dorea* were increased. *Lachnospiraceae* and *Dorea* are genera belonging to Firmicutes and *Lachnospiraceae* produces short-chain fatty acids (SCFAs), especially butyrate, which is not only an important energy source for colonic epithelial cells to maintain normal intestinal barrier function but also a controller in the procedure of intestinal inflammation and the maturation of immune system [27]. At the species level, the abundance of *Bacteroides xylanisolvens Bacteroides*, *Unclassified Lachnospiraceae*, *Bacterium Dorea*, *Bacterium Butyricicoccus*, *Dorea formicigenerans ATCC 27755*, *Unclassified Fusobacterium* and *Unclassified Bifidobacterium* were increased after treatment. The *Unclassified Lachnospiraceae*, *Bacterium Dorea*, *Bacterium Butyricicoccus* and *Dorea formicigenerans ATCC 27755* are species belonging to Firmicutes phylum. In addition, some decreased bacteria such as *Ruminococcaceae NK4A214 group*, *unclassified Ruminococcaceae* and *Peptoniphilus* at the genus level, as well as *Unclassified NK4A214 group*, *Unclassified Ruminococcaceae*, *Gut metagenome Roseburia*, *Unclassified Oscillibacter* and *Uncultured organism Oribacterium* at the species level also belong to Firmicutes. It was noticeable that most altered microbiota after Golden bifid treatment belong to Firmicutes. Therefore, although there were no significant differences of Firmicutes after treatment, we thought that the improvement of symptoms in IBS-D patients might be partially attributed to the relative alteration of Firmicutes.

Interestingly, the abundances of *Bifidobacterium Longum, Lactobacillus bulgaricus* and *Streptococcus thermophilus* did not increase after Golden bifid supplementation. Similarly, Martin et al. [28] found that a 6-month probiotic administration did not significantly alter gut microbiota community structure or diversity as compared to placebo, which might indicate that the improved symptoms of IBS-D patients after probiotics supplementation were not only due to the abundance of probiotics itself has altered, but also accompanied by some other notable changes in the internal environment. For example, Zhou et al. [22] have reported that *Bifidobacterium Longum* alleviated visceral hypersensitivity in rats subjected to water avoidance stress (WAS) by enhancing lysozyme production and promoting mucosal repair. In addition, IBS subjects have increased inflammatory cytokines in gut mucosa compared with controls and suggestive of inflammation in IBS [29]. Golden bifid could alleviate or inhibit further inflammation by suppressing the expression of toll-like receptor 4 (TLR4) [30] and *Bifidobacterium infantis 35624* increased the anti-inflammatory interleukin-10 (IL-10)/proinflammatory interleukin-12 (IL-12) ratio [31]. In terms of the metabolism of gut microbiota, no significant differences of carbohydrate metabolism, amino acid metabolism, nucleotide metabolism, energy metabolism, vitamins metabolism and glycan metabolism were found in fecal microbiota before and after Golden bifid treatments in our study. However, Kim et al. [32] found that probiotics could significantly change metabolites including palmitic acid methyl ester (PAME) and 4,6-dihydroxyquinoline, 4-(2-aminophenyl)-2,4-dioxobutanoic acid (DOBA). Another study showed that *Lactobacillus acidophilus NCFM* and *Bifidobacterium lactis Bi-07* supplementation increased levels of glutamate, SCFAs and ketone bodies and decreased levels of choline, creatine and lactate in weaned rats [33]. These results suggest that probiotic supplementation might alleviate IBS by changing the intestinal environment.

There are several limitations in this study. Firstly, due to the lack of placebo group and a relatively small sample size, the conclusion of this study should be drawn cautiously. Secondly, we found that there were some alterations of microbiota in fecal microbiota of IBS-D patients after Golden bifid treatments. And we inferred that these alterations were associated with the improvement of symptoms IBS-D patients, but further experiments are lacking to confirm this conclusion. Thirdly, no significant differences of microbiota metabolism were found in IBS-D patients before and after Golden bifid treatment and more precise method should be adopted to further explore the effects of Golden bifid on microbiota metabolism.

In conclusion, a 4-week treatment of Golden bifid could significantly improve the abdominal pain, abdominal distension, diarrhea, defecatory urgency, incomplete evacuation and depression symptoms of IBS-D patients. Besides it, after Golden bifid treatment, the abundance of some gut bacteria such as *Lachnospiraceae* and *Dorea* have altered in fecal microbiota, which might imply that the Golden bifid could improve IBS-D via microbiota modulation and provide some evidences for the treatments of probiotics in IBS-D. However, the precise strains and mechanisms of probiotic improve the IBS-D are not fully understood and further studies are warranted to define those and provide more evidences for probiotics treatment of IBS.

## Supplementary Information

**Additional file 1.** Figure S1.

## Data Availability

The datasets used and analyzed during the current study are available from the corresponding author on reasonable request.

## Abbreviations

IBS: Irritable bowel syndrome
IBS-D: Diarrhea-predominant irritable bowel syndrome
GI: Gastrointestinal
SDS: Self-Rating Depression Scale
SAS: Self-Rating Anxiety Scale
SIBO: Small intestine bacterial overgrowth
FBDs: Functional bowel disorders
LHBT: Lactulose hydrogen breath test
OTUs: Operational taxonomic units
PCoA: Principal coordinates analysis
SCFAs: Short-chain fatty acids
