# Supplementary material for: Golden bifid might improve diarrhea-predominant irritable bowel syndrome via microbiota modulation
Source: J Health Popul Nutr. 2022 May 16;41:21. doi: 10.1186/s41043-022-00302-0 (PMC9109320; doi:10.1186/s41043-022-00302-0)
Supplement: Supplementary file 1 — Additional file 1. Figure S1. [file 41043_2022_302_MOESM1_ESM.docx]

**
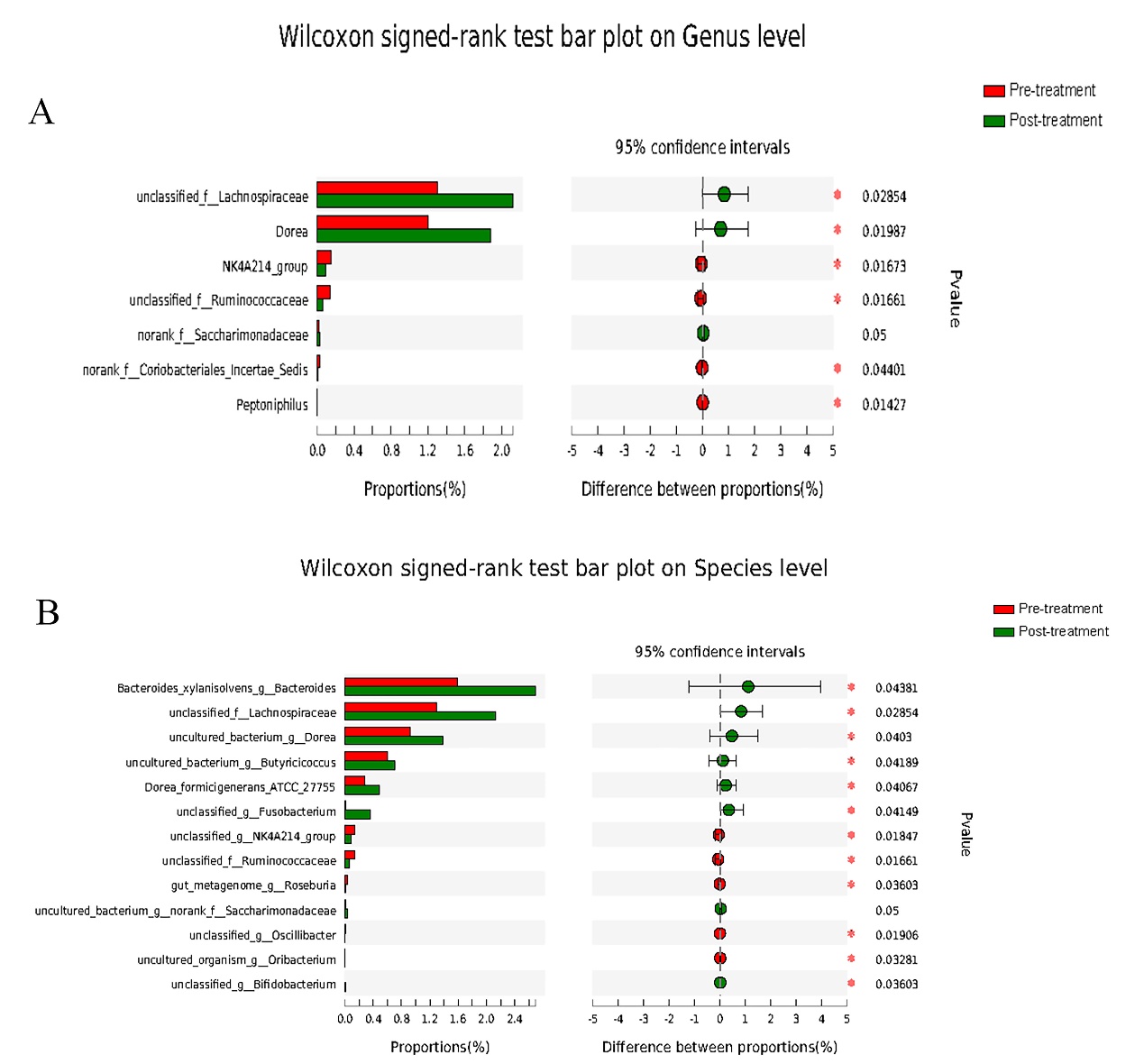
**

**Figure S1.** Comparison of relative abundance of fecal microbiota at genus and species levels. **(A)** Genus levels. **(B)** Species levels.
